# Natural variation in life history strategy of *Arabidopsis thaliana* determines stress responses to drought and insects of different feeding guilds

**DOI:** 10.1111/mec.14100

**Published:** 2017-04-06

**Authors:** Nelson H. Davila Olivas, Enric Frago, Manus P. M. Thoen, Karen J. Kloth, Frank F. M. Becker, Joop J. A. van Loon, Gerrit Gort, Joost J. B. Keurentjes, Joost van Heerwaarden, Marcel Dicke

**Affiliations:** ^1^ Laboratory of EntomologyWageningen UniversityWageningenThe Netherlands; ^2^ Wageningen Plant ResearchWageningen University and ResearchWageningenThe Netherlands; ^3^ Laboratory of Plant PhysiologyWageningen UniversityWageningenThe Netherlands; ^4^ Laboratory of GeneticsWageningen UniversityWageningenThe Netherlands; ^5^ BiometrisWageningen University and ResearchWageningenThe Netherlands; ^6^ Plant Production Systems GroupWageningen UniversityWageningenThe Netherlands

**Keywords:** biotic stress, drought, fungal pathogen, generalist, herbivory, specialist, summer annual, winter annual

## Abstract

Plants are sessile organisms and, consequently, are exposed to a plethora of stresses in their local habitat. As a result, different populations of a species are subject to different selection pressures leading to adaptation to local conditions and intraspecific divergence. The annual brassicaceous plant *Arabidopsis thaliana* is an attractive model for ecologists and evolutionary biologists due to the availability of a large collection of resequenced natural accessions. Accessions of *A. thaliana* display one of two different life cycle strategies: summer and winter annuals. We exposed a collection of 308 European *Arabidopsis* accessions, that have been genotyped for 250K SNPs, to a range of stresses: one abiotic stress (drought), four biotic stresses (*Pieris rapae* caterpillars, *Plutella xylostella* caterpillars, *Frankliniella occidentalis* thrips and *Myzus persicae* aphids) and two combined stresses (drought plus *P. rapae* and *Botrytis cinerea* fungus plus *P. rapae*). We identified heritable genetic variation for responses to the different stresses, estimated by narrow‐sense heritability. We found that accessions displaying different life cycle strategies differ in their response to stresses. Winter annuals are more resistant to drought, aphids and thrips and summer annuals are more resistant to *P. rapae* and *P. xylostella* caterpillars. Summer annuals are also more resistant to the combined stresses of drought plus *P. rapae* and infection by the fungus *Botryris cinerea* plus herbivory by *P. rapae*. Adaptation to drought displayed a longitudinal gradient. Finally, trade‐offs were recorded between the response to drought and responses to herbivory by caterpillars of the specialist herbivore *P. rapae*.

## Introduction

Biotic and abiotic conditions vary in space and time. As a result, different populations of a species are exposed to different selection pressures leading to adaptation to local conditions and intraspecific divergence (Kaltz & Shykoff 1998; Manel *et al*. 2003; Kawecki & Ebert 2004). Locally adapted populations are, therefore, expected to outperform non‐native populations (Kawecki & Ebert 2004; Savolainen *et al*. 2013). Local adaptation is particularly relevant in the face of the global changes our planet is exposed to, because locally adapted populations can become maladapted if environmental conditions change. Mathematical models indicate that climate change will increase the incidence of extreme temperatures, drought and flooding events (Zhao & Running 2010; Sheffield *et al*. 2012; Wheeler & von Braun 2013). With global increase in mean temperatures, it is expected that species will move towards higher elevation and latitudes (Devictor *et al*. 2012). This will result in changes in species interactions and the intensity of selection pressure that biotic and abiotic conditions impose on organisms (Hughes 2000). Thus, understanding how organisms adapt to environmental conditions and investigating how responses to environmental stresses covary with geographic gradients are central issues in exploring the ecological consequences of global change.

There is a wealth of experimental evidence for local adaptation in plants and animals (Bradshaw & Holzapfel 2001; Savolainen *et al*. 2007; Leimu & Fischer 2008; Hereford 2009; Leinonen *et al*. 2009, 2011; Zust *et al*. 2012; Sternberg & Thomas 2014). Local adaptation has been demonstrated along longitudinal, latitudinal and elevational gradients in several plant species (Mikola 1982; Olsson & Agren 2002; Stinchcombe *et al*. 2004; Montesinos‐Navarro *et al*. 2012; Alberto *et al*. 2013; Debieu *et al*. 2013). Important variables such as temperature and precipitation covary with these gradients, and thus, the latter are useful indicators of species adaptation to local environmental conditions (Fukami & Wardle 2005; Walker *et al*. 2010; Halbritter *et al*. 2013; Sundqvist *et al*. 2013; Manzano‐Piedras *et al*. 2014). Traits involved in interactions between organisms and their environment have also been observed to display variation across gradients. For example, tolerance to abiotic stresses such as drought, salinity and low temperature is known to vary with elevation and latitude (Baxter *et al*. 2010; Hancock *et al*. 2011; Alberto *et al*. 2013; Paccard *et al*. 2014). Resistance to biotic stresses such as insect herbivory is also known to vary along geographic gradients. Evidence suggests that herbivory is more intense at lower latitudes and elevations, due to increased herbivore diversity and, hence, plants will be under selection for increased defences (Sanders *et al*. 2003; Andrew & Hughes 2005; Moles *et al*. 2011; Halbritter *et al*. 2013; Sundqvist *et al*. 2013; Rasmann *et al*. 2014). A study on two piperaceous plant species, for example, reported higher incidence of herbivory towards the equator (Salazar & Marquis 2012). Similarly, ecotypes of *Plantago lanceolata* and *Vicia sepium* from low elevations were more resistant to herbivory than plants from high‐elevation ecotypes (Pellissier *et al*. 2014). Although the hypothesis that ‘the lower the latitude and elevation, the higher the level of plant defences’ (Andrew & Hughes 2005; Moles *et al*. 2011) has been supported in some studies, others failed to provide support. Therefore, further experiments are needed that address plant responses throughout extensive geographic areas. As firmly rooted organisms, plants are exposed to a wide diversity of biotic and abiotic stresses which commonly occur simultaneously (Atkinson & Urwin 2012; Kissoudis *et al*. 2014; Suzuki *et al*. 2014). Much progress has been made in understanding how plants adapt to their environment and what the underlying genetic mechanisms are. Yet, we still know rather little about how plants integrate responses to multiple simultaneous stresses, and how this is shaped by local adaptation.

Several methodologies have been developed to investigate local adaptation, including reciprocal transplants and common garden experiments (Kawecki & Ebert 2004). When screening populations from a large geographic area, however, the former approach is often impractical for legal, logistic or ethical reasons, and hence, growing different genotypes in a common environment is a good alternative. Under these circumstances, environmental effects can be removed and variation in phenotypic responses can be assessed. This approach is particularly useful because it allows to investigate the role of a particular agent as driver of population differentiation (Kawecki & Ebert 2004; Alberto *et al*. 2013; Manzano‐Piedras *et al*. 2014). Despite the limitations of inferring local adaptation from correlative approaches in a common environment (Savolainen *et al*. 2013), this method represents a powerful tool for detecting selection along environmental gradients (Gomaa *et al*. 2011; Montesinos‐Navarro *et al*. 2011, 2012; Pico 2012; Debieu *et al*. 2013; Manzano‐Piedras *et al*. 2014).

Model organisms like *A. thaliana* have become attractive to ecologists and evolutionary biologists because large collections of natural accessions with known genomic sequences are available (Shindo *et al*. 2007; Fournier‐Level *et al*. 2011; Weigel 2012). Furthermore, recent studies have demonstrated the feasibility of using these natural accessions for genomewide association studies, allowing to relate variation in a trait with variation in the genes underlying that trait (Atwell *et al*. 2010; Weigel 2012; Bac‐Molenaar *et al*. 2015; Thoen *et al*. 2017). In *A. thaliana*, variation in several life history traits along latitudinal and altitudinal gradients has been demonstrated, including (i) variation in flowering time, seed dormancy and growth rate (Stinchcombe *et al*. 2004; Montesinos‐Navarro *et al*. 2011; Debieu *et al*. 2013); (ii) variation in resistance against abiotic stresses such as salt and drought stress (McKay *et al*. 2003; Baxter *et al*. 2010; Juenger 2013; Easlon *et al*. 2014); and (iii) variation in defence‐related traits. For example, *A. thaliana* displays a latitudinal and longitudinal gradient for diversity in glucosinolate profiles, a main class of defensive metabolites in the Brassicaceae (Brachi *et al*. 2015) and, through a longitudinal gradient, this correlates with the abundance of two aphid species, that is *Brevicoryne brassicae* and *Lipaphis erysimi* (Zust *et al*. 2012).

An important characteristic of *A. thaliana* is that accessions exhibit one of two different life cycle strategies: summer and winter annuals (Pigliucci 1998; Koornneef *et al*. 2004; Shindo *et al*. 2007). Winter annuals are accessions that germinate in late summer and autumn, overwinter as rosette and flower in spring. Winter annuals require a vernalization period to flower. On the other hand, summer annuals or rapid cyclers germinate in spring, flower in summer and do not require vernalization for flowering (Koornneef *et al*. 2004; Stinchcombe *et al*. 2004; Shindo *et al*. 2007). Furthermore, the winter annual life cycle strategy is mostly expressed in temperate areas, whereas the summer annuals occur in warmer regions (Johanson *et al*. 2000; Michaels *et al*. 2003). This plasticity in life cycle strategies has been hypothesized to be the basis for the highly adaptive capacity of *A. thaliana* (Shindo *et al*. 2007). For instance, there is a strong positive genetic correlation between flowering time and water‐use efficiency such that late‐flowering accessions display higher water‐use efficiency than early‐flowering ones (McKay *et al*. 2003; Juenger 2013; Easlon *et al*. 2014). Despite the different phenological, morphological and stress‐response traits observed in *A. thaliana* accessions displaying different life cycle strategies, these differences have rarely been considered when studying local adaptation and responses to abiotic and biotic stresses.

The overall goal of this study was to address this knowledge gap. We have investigated whether accessions of the two *Arabidopsis* life history strategies differ in resistance to a range of biotic and abiotic stresses. We used a collection of 308 European *A. thaliana* accessions and exposed them in a controlled environment to a diverse range of abiotic and biotic stresses.

Phytohormones are major players in the signalling network underlying responses to both biotic and abiotic stresses (Pieterse *et al*. 2009). For instance, chewing insect herbivores elicit especially the jasmonic acid (JA), abscisic acid (ABA) and ethylene (ET) signalling pathways; phloem‐sucking insects and biotrophic microbial pathogens elicit especially the salicylic acid (SA) pathway; and drought elicits the abscisic acid (ABA) pathway (Pieterse *et al*. 2009). These phytohormonal responses exhibit extensive crosstalk, resulting in specific changes in plant phenotype in response to individual stresses (De Vos *et al*. 2005; Pieterse *et al*. 2009). As biotic stresses, we selected four insect herbivores that induce different phytohormonal pathways so as to cover a range of stress types. The herbivores used in this study are two leaf‐chewing caterpillars, *Pieris rapae* and *Plutella xylostella*, and two piercing‐sucking insects, the phloem‐feeding aphid *Myzus persicae* and the cell‐content‐feeding thrips *Frankliniella occidentalis*. Moreover, stress combinations were imposed by combining drought plus *P. rapae* and the necrotrophic fungus *Botrytis cinerea* plus *P. rapae*. *Arabidopsis thaliana*'s response to *P. rapae*,* P. xylostella*,* F. occidentalis*,* B. cinerea* and drought as single stresses is highly diverse, yet at the same time regulated by the plant hormones JA and/or ABA (De Vos *et al*. 2005; Broekgaarden *et al*. 2007; Verhage *et al*. 2011). In contrast, the response to the aphid *M. persicae* is regulated by the plant hormone SA (De Vos *et al*. 2005).

The main goal of this study was to investigate whether winter and summer annuals differ in their responses to a set of different stresses, including biotic and abiotic stresses and some stress combinations. To further assess the basis for the expected differential responses, we also addressed the following questions for the population of *Arabidopsis* accessions that we used here: (a) Is there heritable genetic variation in *A. thaliana’*s responses to the different stresses studied? (b) Does the proportion of winter and summer annuals vary along latitude, longitude and elevation? (c) Do *A. thaliana* plants from different latitudes, longitudes and elevations differ in their responses to these stresses? Life history theory predicts that for a specific genotype, the expression of a particular defensive or resistance trait will constrain the expression of others, so we finally asked the following: (d) Are there trade‐offs among *A. thaliana* responses to different stresses? To identify candidate genes underlying the responses of winter annuals vs. summer annuals, we carried out a multitrait genomewide association analysis.

## Materials and methods

### *Arabidopsis thaliana* populations and molecular markers

In this study, we used a population of 350 *Arabidopsis thaliana* (L.) Heynh. accessions from the Hapmap population (http://bergelson.uchicago.edu/wp-content/uploads/2015/04/Justins-360-lines.xls) (Fig. S1, Supporting information). To avoid geographic outliers, however, our analyses were limited to the data on the European accessions found at a latitude ≥30, longitude between −50 and 50 and elevation ≤2000 m (Fig. S2, Supporting information). This resulted in a subset of 308 accessions on which all further analyses were performed. The Hapmap population has been genotyped for 250K bi‐allelic SNPs (Baxter *et al*. 2010; Platt *et al*. 2010; Chao *et al*. 2012), and after quality control and imputation, this SNP set was reduced to a set of 214 051 SNPs. To get a deeper understanding of these correlates, we obtained a set of environmental variables from the locations where the *A. thaliana* accessions had been collected (Data S1, Supporting information). Sources of these environmental data are summarized in Table S1 (Supporting information). The environmental variables were then correlated with latitude, longitude and elevation through Spearman correlation tests (Fig. S3, Supporting information).

### Genetic diversity in European accessions from the Hapmap population

Determination of geographically informative genetic groups was done as follows. Principal component analysis was performed on the scaled 0,1 molecular marker matrix of dimensions *n* × *m*, where *n* is the number of individuals and *m* the number of markers (van Heerwaarden *et al*. 2011, 2013; Odong *et al*. 2013). Spatial autocorrelation of individual PCs was determined by Moran's I statistic. Geographic coordinates were converted to spatial weight classes by the functions *graph2nb* and *nb2listw* in the R package *spdep*. The first *k* principal components with the highest spatial autocorrelation and associated *P* values below 0.001 were retained as spatially informative and were used to calculate a Euclidean distance matrix. A dendrogram was produced by Ward clustering, and genetic groups were assigned by splitting the dendrogram into groups using the R function *cutree*. Phenotypic differences between genetic groups were tested by ANOVA after correction for life cycle strategy.

### Classification of accessions as winter and summer annuals

Flowering time and flowering time after vernalization under greenhouse conditions have been recorded for this plant population as reported by Bac‐Molenaar *et al*. (2016). Accessions were classified as winter annuals if vernalization was required for flowering (flowering time ≥75 days) or as summer annuals otherwise (Data S1, Supporting information).

### Plants, insects and fungi

*Arabidopsis thaliana* plants were grown in a controlled environmental chamber at 24 ± 1 °C, 70 ± 10% relative humidity, 200 μmol m^−2^ s^−1^ photosynthetically active radiation from fluorescent lights (TL‐D 58W/840; www.philips.com) and a diurnal cycle of 8:16 L:D. In all experiments, seeds were vernalized at 4 °C for 5 days to induce even germination. Plants were individually grown in 0.08 L pots (http://uk.poeppelmann.com/teku/home/) in a pasteurized (4 h at 80 °C) commercial *Arabidopsis* potting soil, which was mixed 1:1 (v/v) with autoclaved sand in experiment (1). In experiments (2), (3) and (4) (see later), plants were grown in pasteurized (4 h at 80 °C) commercial potting soil. Individual pots were accommodated in trays, randomly distributed within the growth chamber. Plants were watered three times per week by adding water to the tray. Once per week, they received *Steinernema feltiae* entomopathogenic nematodes (Entonem; http://www.koppert.nl/) to prevent infestation by fungus gnats.

*Pieris rapae* L. (Small Cabbage White butterfly; Lepidoptera; Pieridae) were reared on Brussels sprouts plants (*B. oleracea* var. *gemmifera* cv Cyrus) in a growth chamber at 21 ± 1 °C, 50–70 % relative humidity and a diurnal cycle of 16:8 L:D.

*Plutella xylostella* L. (Diamondback Moth; Lepidoptera; Yponomeutidae) were reared on Brussels sprouts plants (*B. oleracea* var. *gemmifera* cv Cyrus) in a growth chamber at 22 ± 1 °C, 40–50% relative humidity and a diurnal cycle of 16:8 L:D.

The Western flower thrips (*Frankliniella occidentalis* (Pergande)) used in this study were originally collected from *Chrysanthemum* flowers and reared in glass bottles on green common bean pods (*Phaseolus vulgaris*) in a climate cabinet at 25 ± 1 °C, 50–70% relative humidity and a diurnal cycle of 16:8 L:D.

*Myzus persicae* (Sulzer) (Green Peach Aphid; Hemiptera: Aphididae) were reared on radish plants, *Raphanus sativus* L., at 19 ± 2 °C, 50–70% relative humidity and a diurnal cycle of 16:8 L:D.

The necrotrophic fungus *Botrytis cinerea*, strain B0510 (Van der Ent *et al*. 2008), was grown on half‐strength PDA plates containing penicillin (100 μg mL^−1^) and streptomycin (200 μg mL^−1^) for 2 weeks at room temperature. Spores were collected and resuspended in half‐strength potato dextrose broth (Difco Laboratories) to a final density of 1.0 × 10^5^ spores mL^−1^. After a 3‐h incubation period, the spores were used for inoculation (Thomma *et al*. 1998; Pre *et al*. 2008; Van der Ent *et al*. 2008).

### Experimental design and treatments

We performed four different experiments in which plants were exposed to the following stresses (Fig. S4, Supporting information): experiment (1): drought, damage by *P. rapae* caterpillars alone or preceded by drought, or infestation by *B. cinerea*; experiment (2): damage by *P. xylostella* caterpillars; experiment (3): damage by the cell‐content‐feeding thrips *F. occidentalis* and experiment (4): infestation by the phloem‐feeding aphid *M. persicae*.

In experiment (1), plant responses were quantified for all 308 accessions with six replicates per accession. Bioassays were performed in 10 temporal blocks. Each block included 28–33 random accessions. To correct for variation among temporal blocks, a fixed set of three accessions (CS28780; Tsu‐0, CS76113; Col‐0 and CS76129; Fei‐0) was included in each temporal block. The spatial location of each plant was recorded. Within temporal blocks, plants were allocated in trays and the position of the tray in the rearing chamber recorded as its position in either of the six racks, each with four shelves. The position of each plant in the trays was also recorded in terms of X and Y coordinates. In each temporal block, accessions were exposed to the following five treatments with a total of six replicates per accession and treatment: (a) no stress, (b) drought stress, (c) *P. rapae* herbivory, (d) drought plus *P. rapae* herbivory or (e) *B. cinerea* infection plus *P. rapae* herbivory (Fig. S4A, Supporting information). Plants were grown under similar conditions during the first 3 weeks. Drought stress was imposed by withholding water for 7 days during the third week, while the rest of the plants was watered every 2 days with 1 L of water per tray. Withholding water for 7 days resulted in plants that displayed retarded growth and were smaller than well‐watered plants. *Botrytis cinerea* inoculation was carried out 24 h prior to *P. rapae* inoculation. Plants were inoculated with *B. cinerea* by pipetting 5 μL of spores suspended in half‐strength PDB (Difco Laboratories) at a concentration of 1 × 10^5^ spores mL^−1^ onto two of the leaves in the rosette. To ensure successful infection by *B. cinerea*, plants were kept at 100% RH for 24 h. Four‐week‐old plants were exposed to herbivory by *P. rapae* as single or combined stress. Infestation with this species was carried out by placing two newly hatched first instar (L1) caterpillars on one of the leaves; they were allowed to feed for 5 days. To prevent caterpillars from moving between plants, individual plants were placed on the inverted lid of a Petri dish and trays were filled with water at the moment of inoculation. While plants were on the Petri dishes, they received the same watering regime as described above; however, watering was carried out by adding 20 mL of water to each Petri dish. Subsequently, plant rosette fresh weight was quantified for all treatments.

In experiment (2), all 308 accessions were used. However, some of the accessions displayed some germination problems so we did not have enough replicates for the experiment; this reduced the data set from 308 to 265 accessions. Four replicates were used for each of these accessions. The bioassays were performed in four temporal blocks. In each block, all accessions were phenotyped; one replicate per accession per temporal block, so block is the level of replication. Thus, the four temporal blocks resulted in four replicates per accession. Within blocks, accessions were randomly distributed over 40 trays. In this experiment, accession Col‐0 was included to control for possible positional effects within the chamber. Each tray contained both control and treatment plants for Col‐0 and the other accessions. Plant positions were randomized within the trays. Within each block, accessions were exposed simultaneously to either (a) no stress or (b) herbivory by *P. xylostella*. Plants were 4 weeks old when they were inoculated with two L2 larvae. Larvae were allowed to feed for 5 days. To prevent caterpillars from moving between plants, the same procedure was used as described above for experiment (1). Subsequently, plant rosette fresh weight was quantified for all treatments (Fig. S4B, Supporting information).

In experiment (3), bioassays were carried out for all 308 accessions with five replicates per accession. Bioassays were performed in five temporal blocks. Within each block, one replicate per accession was screened and so block is the level of replication. Leaves from 5‐week‐old plants were cut (one leaf per plant) and transferred to Petri dishes (diameter 5 cm; BD falcon, Product Number: 351006) containing a film of 1% agar. The petiole was inserted into the agar film (Fig. S4C, Supporting information). Leaves were exposed to three juvenile (second‐larval instar, L2) *F. occidentalis* for 6 days (Fig. S4C, Supporting information). Feeding damage was estimated in mm^2^ after 6 days by counting the number of small ‘silver damage’ feeding spots, where one small feeding spot accounts for 3 mm^2^ damage (bigger spots were counted as 2–5 small spots).

In experiment (4), bioassays were carried out for all 308 accessions with two to three replicates per accession. The experiment was performed in three temporal blocks, each divided into four sub‐blocks, representing four consecutive days. Three‐week‐old plants were inoculated with one 0–24 h‐old *M. persicae* nymph per plant. Individual plants were placed on the inverted lid of a Petri dish in trays with a soap‐diluted water barrier to prevent aphids from moving between plants. Each tray contained 16 plants. Fourteen days after infestation, the number of aphids per plant was counted (Fig. S4D, Supporting information).

The bioassay data are part of plant response data collected for a global population of 350 accessions. The data for this global population have been used previously to investigate the genetic architectures underlying single and combined stress responses (Davila Olivas *et al*. 2017; Thoen *et al*. 2017). Here, we used the stress‐response data of only the 308 European accessions to specifically investigate differences in stress responses between summer annuals and winter annuals.

### Statistical analysis

#### Genotypic means

For each genotype, we estimated a genotypic mean response to each stress (Jimenez‐Gomez *et al*. 2010; Filiault & Maloof 2012; Riedelsheimer *et al*. 2012). These estimated means (or predicted values) were extracted from linear mixed effect models that were fitted with the ASReml package in R (Harrell 2009).

Experiment 1: *Y *= *μ +* *GEN + TRT + GEN* × *TRT + *random terms + e,
Experiment 2: *Y *= *μ +* *GEN + TRT + GEN* × *TRT +* random terms + e,
Experiment 3: *Y = μ + GEN + *random terms + e,
Experiment 4: *Y = μ + GEN + *random terms + e.

In all these models, *Y* represents the measured variable in each experiment (i.e. *A. thaliana* rosette fresh weight, thrips silver damage or aphid reproduction), and e is the residual error. TRT is the treatment factor and *GEN* is genotype (accession) which were included as fixed factors. Random terms were included to correct for temporal and positional effects. Below, B refers to blocks, SB to sub‐blocks, R to racks, S to shelves, T to trays, X to *x*‐coordinate within tray and Y to *y*‐coordinate within tray. The random part of experiment 1 consisted of random terms for B, R, S, B × R × S, B × R × S × T, B × R × S × T × X, and B × R × S × T × Y. For experiment 2, it consisted of random terms for B, B × T, B × T × X and B × T × Y, and for experiments 3 and 4, random terms for B and B/SB. In the first two experiments, predicted means from the fitted models were used to estimate variables that capture the response to each stress. The response to each stress consisted of *A. thaliana* fresh weight in the form of percentage reduction relative to control plants not exposed to stress. In the treatment where plants were exposed to both drought and herbivory by *P. rapae*, percentage weight reduction was relative to plants that experienced drought. From here onward, we will refer to the response to each stress as *P. rapae*, Drought, Drought&*Pieris*,* Botrytis*&*Pieris* and *P. xylostella*, respectively. Feeding damage by thrips will be referred to as *F. occidentalis* and number of aphids as *M. persicae*. These variables are summarized in Data S1 (Supporting information). Furthermore, these variables were used in downstream analyses as response variables in phylogenetic mixed models and to estimate heritability as explained in the section below.

#### Phylogenetic mixed models, heritability estimations

When comparing traits among species or among different populations of the same species, one needs to control for the nonindependence of data points (correlated residuals) due to shared ancestry. This may be achieved by statistical methods such as phylogenetic mixed models or animal models (Hadfield 2010; Hadfield & Nakagawa 2010). A phylogenetic mixed model is a type of mixed effects model where, in addition to any other fixed or random effects, a pedigree representing the genealogy of the individuals is included in the model. This genealogy is then transformed into a variance–covariance matrix of relatedness between individuals and included in the model as a random additive genetic effect (Kruuk, 2004; Postma & Charmantier, 2007). In our study, such genealogy was based on the kinship matrix obtained from the 214 051 *A. thaliana* SNPs (Atwell *et al*. 2010) as is common in genetic association studies (Kim *et al*. 2007; Li *et al*. 2010; Bac‐Molenaar *et al*. 2015). By doing so, we accounted for the nonindependence of plant phenotypic responses among genetically similar plant accessions. This is particularly relevant, for example when specific genotypes aggregate in a particular geographic location not because of the action of natural selection but because of genetic drift. To test hypotheses about the effect of plant life cycle strategy and geographic location on plant responses to stress, we built a different phylogenetic mixed model for each stress. Phenotypic responses of the plants were included as response variable with a Gaussian distribution. As predictors we included (i) plant life cycle strategy as a categorical variable (winter vs summer annual) and (ii) latitude, (iii) longitude and (iv) elevation as continuous variables (phylogenetic model type 1). The effect of environmental gradients (latitude, longitude and elevation) on *A. thaliana* plant life cycle strategy was also modelled with similar phylogenetic mixed models but with plant life cycle strategy included as a categorical response variable (phylogenetic model type 2). To explore potential trade‐offs among plant responses to different stresses, a different phylogenetic mixed model was also built for each of the single stresses studied here. In each phylogenetic mixed model, we included plant responses to a particular stress as a response variable with a Gaussian distribution, and as predictors, we included responses to the other stresses and plant life cycle strategy (phylogenetic model type 3). All phylogenetic mixed models were fitted with Bayesian Markov chain Monte Carlo (MCMC) techniques implemented in the MCMCglmm package in R (Hadfield 2010; Hadfield & Nakagawa 2010). In the phylogenetic mixed models types 1 and 3, the MCMC chain ran for 150 000 iterations. To prevent autocorrelation among subsequent iterations, the chain was sampled every 50 iterations with the first 50 000 removed as burn‐in. In the phylogenetic mixed model type 2, all MCMC parameters where ten times larger so that the chain ran for 1.5 million iterations. Autocorrelation between consecutive values was always lower than 0.1, and convergence of the chains was confirmed by visual inspection so that there were no trends in the chain and posterior distributions were not skewed. Fixed effects are presented as the posterior mean (PM) with the credible intervals (CI) of the estimate, with significance reported as the pMCMC statistic (Hadfield 2010; Hadfield & Nakagawa 2010). In a Bayesian model, probability distributions need to be specified via specific priors for the fixed effects and the covariance matrices. Because we did not have any a priori knowledge on the distribution of our data, we used flat priors. For phylogenetic mixed models type 1 and 3, we used priors with scale = 0.002 and degree of belief = 0.002 (i.e. *V* = 1, nu = 0.002). For the phylogenetic mixed models type 2, we used an inverse Wishart prior with scale = I*0.002 and degree of belief = 2.002 (i.e. *V* = diag(1)*(0.002/2.002), nu = 2.002). Narrow‐sense heritability for each response was also estimated with the heritability package in R (Kruijer *et al*. 2015).

#### Multi‐Trait Mixed Model (MTMM) Genomewide Association analysis

Two trait vectors were generated based on their response in summer vs. winter annuals (Fig. 2):

*p1*: Traits where summer annuals were more resistant than winter annuals (*P. rapae*,* P. xylostella*, Drought&*Pieris*,* Botrytis*&*Pieris*).
*p*2: Traits where winter annuals were more resistant than summer annuals (*F. occidentalis*,* M. persicae*, Drought).

The first principal component of each vector was used as a phenotype for the MTMM GWA analysis. MTMM was carried out as described by Korte *et al*. (2012), using 214k SNPs with a minor allele frequency >0.05. Briefly, a multiple trait mixed model was fitted for phenotypes of *n* individuals as *Y *= *X*β + *G* + *E*, with *Y* being the genotypes by traits (*n* × *p*) matrix of phenotypic observations. The terms *X*β, *G* and *E* stand for, respectively, the fixed effects (including trait specific intercepts and SNP effects) and the random genetic and environmental effects. *G* follows a zero mean matrix‐variate normal distribution with row–covariance (marker‐based kinship) matrix K and column (trait) covariance matrix *V*_g_. *V*_g_ is a *p *× *p* matrix modelling the genetic correlations between traits. Similarly, *vec*(*E*) follows a zero mean normal distribution with covariance *V*_e_ ⨂ *I*_n_, where *V*_e_ accounts for the nongenetic correlations between traits.

Using MTMM with multiple traits, we can find causal loci with (a) a common effect across traits, (b) trait‐specific loci or (c) loci with opposite effects. For this purpose, MTMM uses a generalized least square (GLS) *F* test. For any two traits, it can be constructed as follows:

$$y=\left[\begin{array}{c} p1\\ p2 \end{array}\right]=s_{1}\mu_{1}+s_{2}\mu_{2}+x\beta +\left(x\times s_{1}\right)+\alpha +\upsilon ,$$

where *x* is the marker, ***s***_**1**_ is a vector of 1 for all values belonging to the first trait and 0 otherwise, and ***s***_**2**_ is a vector of 1 for all values belonging to the second trait and 0 otherwise. ***υ*** (0,cov(*y*)) is a random variable that captures the error and the genetic random effect. MTMM applies the following tests:

(full); The full model tested against a null model where β = 0 and α = 0. This test identifies both loci with common and differing effects in one model but suffers in power from the extra degree of freedom.
(common); A model α = 0 tested against a null model where β = 0 and α = 0. This test identifies loci with common effect.
(specific); The full model tested against a model where α = 0. This test identifies loci specific or with opposite effect.

#### Candidate gene selection

For selection of candidate genes, an arbitrary threshold was considered. Regions containing SNPs with −log_10_(*P*) ≥ 4 were considered for further analysis as described by El‐Soda *et al*. (2015). A search window was defined by a 20‐Kb region around significant SNPs. All genes within a search window were considered to be potential candidate genes. Genes were annotated according to TAIR 10. Relative gene expression data for *A. thaliana* (Col‐0) plants exposed to jasmonic acid or salicylic acid were obtained from http://bar.utoronto.ca/.

## Results

### Population structure of the European accessions of the Hapmap population

**Figure 1:**
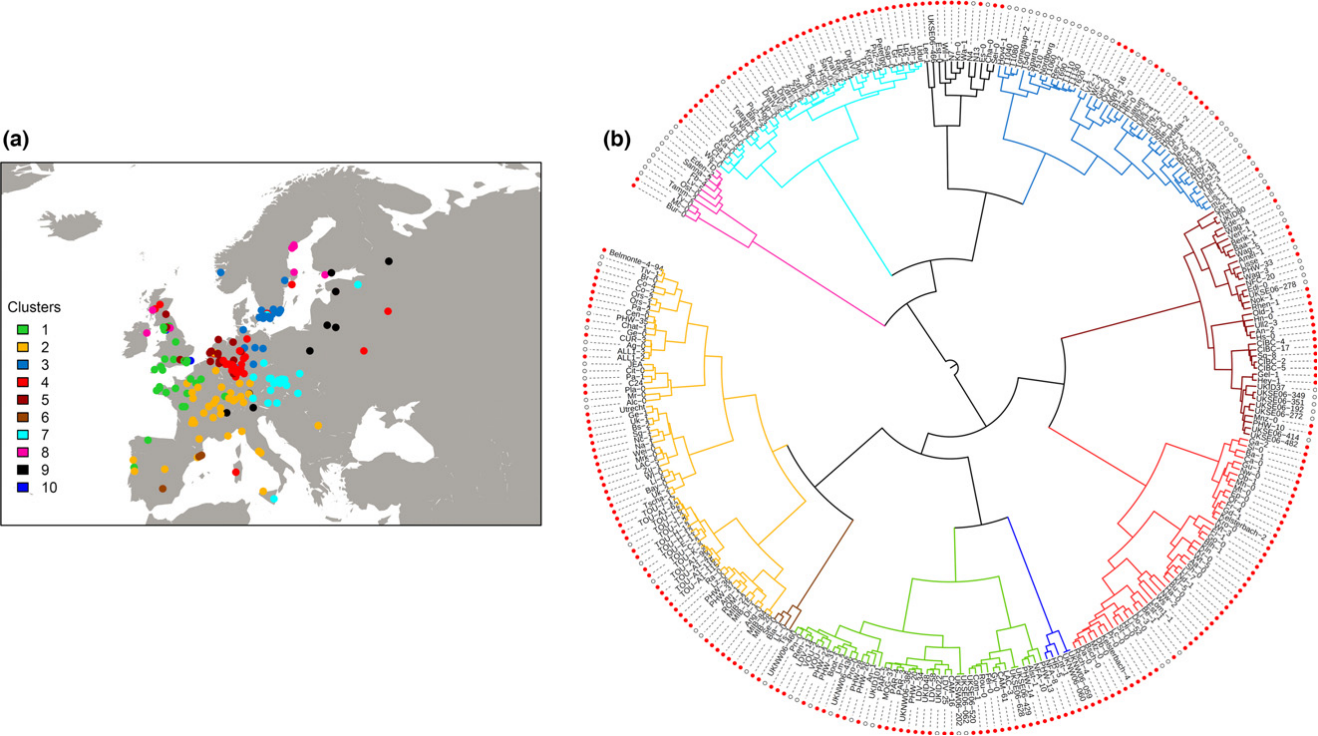
Population structure in the European accessions from the Hapmap population. Clusters of accessions were generated based on kinship matrix. (a) Geographic distribution of genetic clusters is shown in colours. (b) Dendrogram shows the relationship among accessions. Branch colours correspond to the colours in the map. Life cycle strategy is indicated by red circles (summer annuals) and white circles (winter annuals). Dendrogram was generated with itol (http://itol.embl.de/index.shtml).

We first examined the patterns of population structure of the European accessions in the Hapmap population. The first nine genetic principal components, explaining 12% of total variance, showed strong geographic autocorrelation and were used to subdivide the European accessions into ten geographically distinct genetic groups (Fig. 1).

### Heritability of *A. thaliana* responses to abiotic and biotic stresses

An important condition for natural selection to act upon a trait (and therefore to allow local adaptation) is that this trait has phenotypic variation which is genetically determined. This condition was met for *A. thaliana* responses to different stresses as the different traits that we quantified showed substantial heritable variation (Table 1). The largest trait variation was observed for the response to *P. xylostella* (CV = 138%), while the response to *M. persicae* displayed the lowest variation (CV = 20%). No relationship was observed between degree of trait variation and heritability. The largest heritability was observed for feeding damage by thrips (*F. occidentalis*) (*h* ^2^ = 0.90), while plant biomass reduction after *P. xylostella* feeding had the lowest heritability (*h* ^2^ = 0.25). The response to Drought&*Pieris* exhibited less variation and a lower heritability (CV = 33%, *h* ^2^ = 0.39) than the response to *P. rapae* alone (CV = 43%, *h* ^2^ = 0.60). The response to *Botrytis*&*Pieris* (CV = 117%, *h* ^2^ = 0.67) had more variation and a higher heritability than the response to *P. rapae* alone.

**Table 1:** Summary of trait values for *A. thaliana* resistance to abiotic and biotic stresses

Response trait	Experiment	Min.	Mean	Max.	CV (%)	*N*	*h* ^2^	va	ve
Drought^a^	1	−25.94	19.22	50.07	75	308	0.41	83.32	119.96
*P. rapae* a	1	−5.03	32.40	87.80	43	308	0.60	114.79	76.72
Drought&*Pieris* a	1	−3.39	48.25	143.80	33	308	0.39	96.15	153.52
*Botrytis*&*Pieris* a	1	−31.44	14.42	90.13	117	308	0.67	190.83	95.41
*P. xylostella* a	2	−24.86	14.15	78.16	138	265	0.25	94.08	287.25
*F. occidentalis* b	3	0.00	21.98	56.51	43	308	0.90	77.46	8.28
*M. persicae* c	4	13.12	27.89	44.12	20	299	0.34	10.57	20.58

*h* ^2^ = Narrow‐sense heritability, va = additive genetic variance, ve = residual variance, CV = coefficient of variation, *N* = number of observations after subset for European accessions.

a Trait values are expressed in %.

b Trait values are expressed in mm^2^.

c Trait values are counts.

### Effect of life history strategy and genetic structure on stress responses in *A. thaliana*

We classified *A. thaliana* accessions as winter and summer annuals based on their flowering time. Of the 308 accessions analysed, 89 did not produce flowers after 75 days and were therefore classified as winter annuals, while 219 behaved as summer annuals (Data S1, Supporting information). Within this collection of genotypes, winter annuals were more often found in lower elevations and higher latitudes, whereas longitude was not a significant explanatory variable (Fig. S5, Supporting information; Table 2). Furthermore, the two life history strategies were not equally distributed among genetic groups. For example, cluster 8 and cluster 3 had mostly winter annuals while cluster 10 comprised summer annuals exclusively (Figs 1B, S5B, Supporting information). Phenotypic differences between genetic groups were tested by ANOVA after correction for life cycle strategy. Overall, stress responses did not differ significantly between genetic clusters, with the exception of the response to *F. occidentalis* (*P* < 10^−6^) (Fig. S6, Supporting information).

**Table 2:** Bayesian phylogenetic mixed model analysis to assess differences in life cycle strategy and geographic gradients for each stress. For each variable, the posterior mean and 95% credible intervals (in parentheses) are presented. For the fixed effects, the Bayesian *P*‐value is also presented, and significance indicated in bold text

Response trait	Life cycle strategy		Elevation		Latitude		Longitude		Plant genealogy
	PM	*P*	PM	*P*	PM	*P*	PM	*P*	PM
Drought	**−9.21 (−12.59 to −5.62)**	**<0.001**	0.003 (−0.004 to 0.010)	0.389	−0.12 (−0.56 to 0.27)	0.566	**0.229 (−0.004 to 0.426)**	**0.043**	1.51 (<0.001–7.133)
*P. rapae*	**4.61 (1.38–8.18)**	**0.007**	−0.005 (−0.012 to 0.001)	0.167	0.12 (−0.29 to 0.53)	0.575	−0.03 (−0.24 to 0.19)	0.768	4.22 (<0.001–21.26)
Drought&*Pieris*	**7.37 (3.10–11.13)**	**<0.001**	−0.002 (−0.011 to 0.005)	0.534	0.23 (−0.24 to 0.68)	0.352	−0.09 (−0.32 to 0.14)	0.432	2.34 (<0.001–11.81)
*Botrytis*&*Pieris*	**10.68 (6.76–14.81)**	**<0.001**	−0.004 (−0.013 to 0.003)	0.329	0.25 (−0.23 to 0.73)	0.326	−0.11 (−0.38 to 0.12)	0.383	3.05 (<0.001–14.65)
*P. xylostella*	**10.40 (4.86–15.75)**	**<0.001**	0.004 (−0.007 to 0.015)	0.444	−0.05 (−0.74 to 0.58)	0.867	−0.07 (−0.45 to 0.25)	0.683	5.49 (<0.001–28.58)
*F. occidentalis*	**−3.50 (−6.00 to −1.31)**	**0.004**	−0.003 (−0.008 to 0.001)	0.149	0.02 (−0.29 to 0.32)	0.893	0.16 (−0.03 to 0.35)	0.102	38.99 (0.029–99.53)
*M. persicae*	**−1.75 (−3.20 to −0.19)**	**0.018**	−0.001 (−0.003 to 0.002)	0.629	−0.08 (−0.27 to 0.09)	0.357	0.008 (−0.118 to 0.123)	0.851	3.78 (<0.001–17.17)
Flowering type	NA	NA	**−0.26 (−0.55 to −0.01)**	**0.031**	**15.58 (4.21 to 29.39)**	**0.003**	−0.971 (−6.61 to 4.31)	0.732	0.01 (<0.001–0.02)

PM = Posterior mean, *P *= Bayesian *P*‐value. Plant genealogy was included as random effect in the models; thus, *P*‐values are not reported.

**Figure 2:**
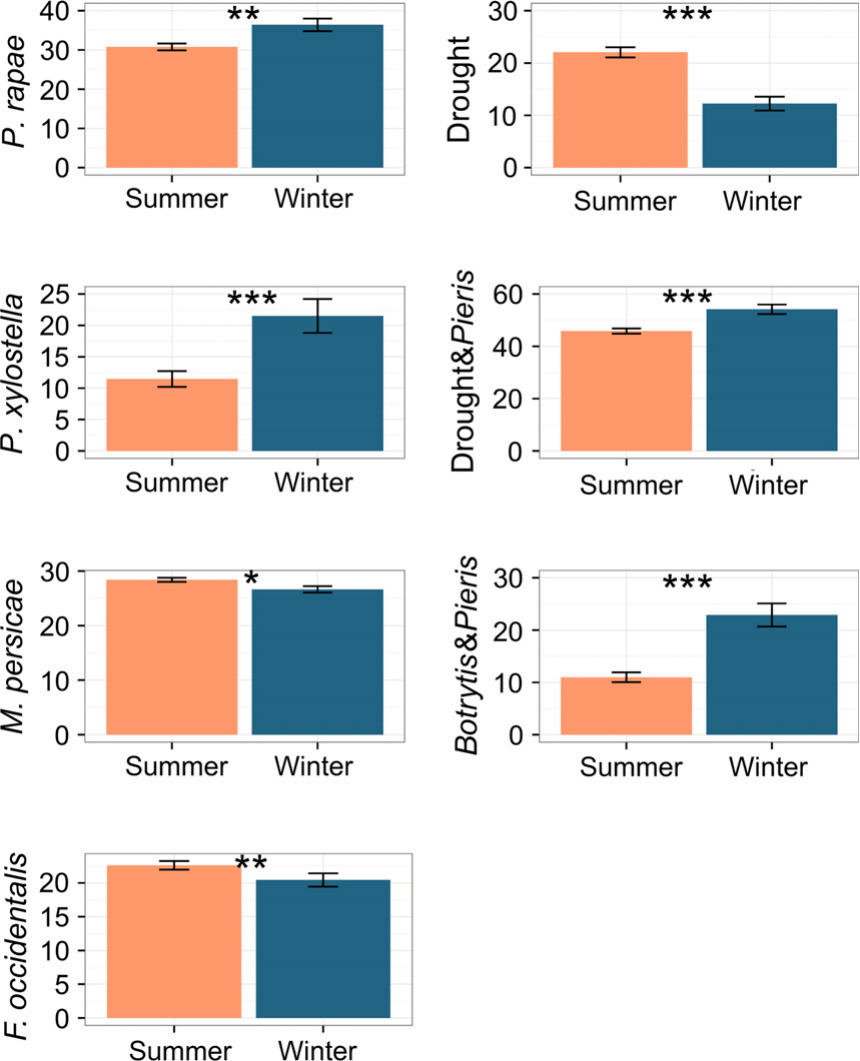
Stress responses of *A. thaliana* accessions belonging to either winter annual or summer annual life cycle strategies. Accessions that required vernalization for flowering were classified winter annuals (blue, *n* = 89); the rest were classified summer annuals (orange, *n* = 219). Bars show mean value ± SE. Stress responses to *P. rapae* caterpillars, *P. xylostella* caterpillars, Drought, Drought&*Pieris* and *Botrytis&Pieris* are represented by the percentage of biomass reduction in *A. thaliana* in response to each stress. Response to *M. persicae* is represented by the number of aphids produced. Response to *F. occidentalis* thrips is represented by the amount of feeding damage in mm^2^. Bayesian *P*‐values are indicated as *P ≤ 0.05, **P < 0.01, ***P < 0.001.

A different phylogenetic mixed model was built for each stress factor, to explore whether plant responses varied between winter and summer annuals and among accessions located along geographic gradients. We found that life cycle strategy significantly affected all responses measured (Table 2; Fig. 2). Summer annuals were more resistant than winter annuals with regard to stresses that included caterpillars. The percentage of *A. thaliana* biomass reduction as a result of feeding by *P. rapae* and *P. xylostella* caterpillars was 6% and 10% larger in winter than in summer annuals, respectively. The percentage of biomass reduction by the combined stresses Drought&*Pieris* and *Botrytis*&*Pieris* was 8% and 12% larger in winter than summer annuals, respectively. Winter annuals were more resistant to drought stress and piercing‐sucking insects than summer annuals. The percentage of biomass reduction in *A. thaliana* caused by drought was 10% lower in winter annuals than in summer annuals. Feeding damage by thrips (*F. occidentalis*) was 8% lower in winter annuals than in summer annuals, and the number of offspring produced by aphids (*M. persicae*) was 6% lower on winter annuals than on summer annuals. In all phylogenetic mixed models for plant responses to stress, and in accordance with our heritability estimates, the importance of *A. thaliana* genealogy was moderate, but relatively higher for the models on plant responses to thrips (*F. occidentalis*; Table 2).

### Effect of environmental gradients on stress responses in *A. thaliana*

We hypothesized that latitude, longitude and elevation of the locations from which the accessions had been collected would affect plant responses and, hence, these variables were also included in the phylogenetic mixed models as predictors. Although the three gradients were highly correlated with several environmental variables measured at each sampling location (Fig. S3, Supporting information), we found only a single significant association with *A. thaliana* responses to stress (Table 2). In particular, we found a significant, positive association between longitude and percentage of biomass reduction caused by drought (Table 2; Fig. S7, Supporting information). Furthermore, a longitudinal gradient was observed for flowering time and a latitudinal gradient was observed for flowering time after vernalization (Table S2, Fig. S7, Supporting information).

### Trade‐offs in *A. thaliana* responses to stress

**Figure 3:**
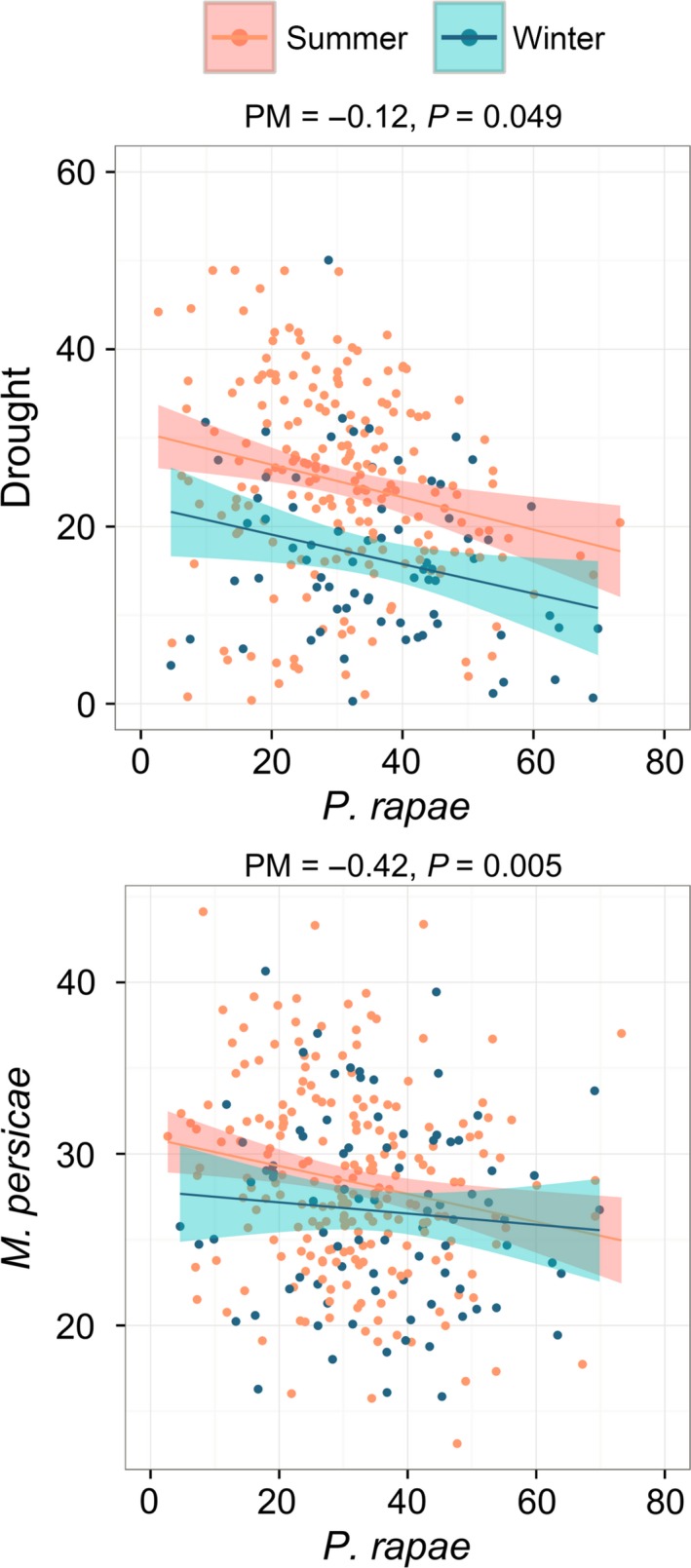
Trade‐off between response to abiotic and biotic stresses among *A. thaliana* accessions. Accessions that required vernalization for flowering were classified winter annuals (blue); the rest were classified summer annuals (orange). Stress responses to *P. rapae* and Drought are represented by the percentage of biomass reduction in *A. thaliana* in response to each stress. Response to *M. persicae* is represented by the number of aphids produced. Lines represent a linear regression fit and shades the 95% confidence interval. These lines were just used for better appreciation of the data because correlations tests were carried out using a linear mixed model as described in Materials and Methods. Posterior means (PM) and (*P*) Bayesian *P*‐values are indicated.

We built a separate phylogenetic mixed model for each of the single stresses and assessed whether they correlated with the other stresses. Because of its importance, plant life cycle strategy was also included as a cofactor in these models. Among the different stresses, we found a clear negative relationship between response to drought and to *P. rapae* herbivory (posterior mean = −0.12, *P *= 0.049), which indicates that these two responses trade‐off (Fig. 3; Table 3). Responses to *P. rapae* herbivory were also negatively correlated with reproduction by the aphid *M. persicae* (posterior mean = −0.42, *P *= 0.005).

**Table 3:** Bayesian phylogenetic mixed model analysis to assess correlations among single stresses. For each variable, the posterior mean, 95% credible intervals and Bayesian *P*‐values are presented. Significance is indicated in bold text

Response trait	Life cycle strategy		Drought		*P. rapae*		*P. xylostella*		*F. occidentalis*		*M. persicae*	
	PM	*P*	PM	*P*	PM	*P*	PM	*P*	PM	*P*	PM	*P*
Drought	**−6.70 (−10.77 to −2.86)**	**<0.001**			**−0.13 (−0.26 to −0.01)**	**0.032**	−0.08 (−0.16 to 0.01)	0.075	0.14 (−0.03 to 0.32)	0.133	0.09 (−0.21 to 0.38)	0.551
*P. rapae*	2.26 (−2.09 to 6.13)	0.276	**−0.12 (−0.24 to 0.01)**	**0.049**			0.05 (−0.03 to 0.14)	0.227	0.12 (−0.06 to 0.30)	0.187	**−0.42 (−0.70 to −0.13)**	**0.005**
*P. xylostella*	7.7 (2.42–13.35)	**0.003**	−0.16 (−0.32 to 0.04)	0.084	0.12 (−0.06 to 0.28)	0.196			−0.15 (−0.40 to 0.10)	0.262	<0.001 (−0.41 to 0.42)	0.988
*F. occidentalis*	**−3.72 (−6.39 to −1.11)**	**0.011**	0.05 (−0.03 to 0.12)	0.198	0.06 (−0.02 to 0.13)	0.153	−0.03 (−0.08 to 0.03)	0.314			−0.05 (−0.25 to 0.13)	0.649
*M. persicae*	−1.09 (−2.93 to 0.43)	0.191	0.01 (−0.03 to 0.07)	0.524	**−0.07 (−0.12 to −0.02)**	**0.002**	<0.001 (−0.04 to 0.04)	0.973	0.01 (−0.08 to 0.08)	0.809		

PM, Posterior mean; *P*, Bayesian *P*‐value.

### Multi‐trait GWA analysis

As our models showed that life history strategy is a predictor for all of the tested stress responses, we explored which genes were associated with flowering‐time‐related resistance by genomewide association mapping. Two trait vectors were generated by taking the first principal component (PC) of stresses that displayed a resistance response in summer annuals (*P. rapae*,* P. xylostella*, Drought&*Pieris*,* Botrytis*&*Pieris*, PC1 = 55%) and the first PC of stresses with a resistance response in winter annuals (*F. occidentalis*,* M. persicae*, Drought, PC1 = 40%).

**Figure 4:**
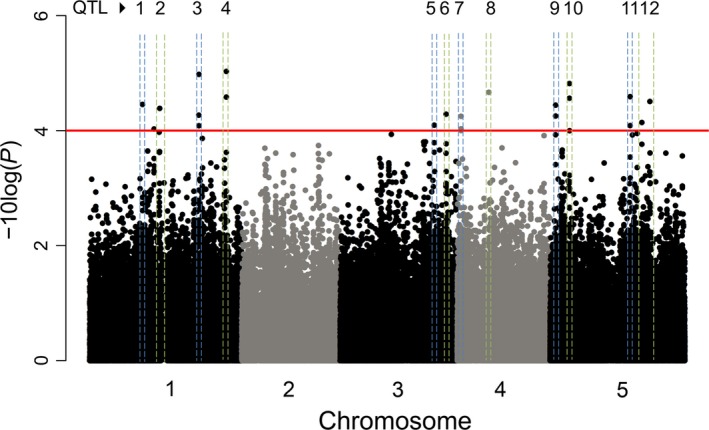
Multi‐trait GWA analysis. Red line indicates an arbitrary threshold set at −log10(*P*) ≥ 4 as described in the Methods section for detecting significant associations. QTL regions are indicated with numbers. Detailed information about each QTL is presented in Data S2 (Supporting information).

MTMM analysis identified a total of 23 trait‐specific SNPs at −log_10_(*P*)‐value ≥4, which were distributed across 12 QTL regions, containing a total of 168 genes, in the genome (Fig. 4, Data S2, Supporting information). These 12 regions (QTLs) contained putative biotic stress‐related genes, such as a pathogenesis‐related thaumatin gene (*At5g40020*), and several life history strategy‐related candidates. Two genes in QTL 6 (*At3g57390*:* AGL18* and *At3g57300*:*INO80*) are members of the flowering‐time gene network (Bouché *et al*. 2016). Interestingly, *ino80* mutants display a late‐flowering phenotype, while the *agl18agl15* double mutant displays an early‐flowering phenotype (Fernandez *et al*. 2014; Zhang *et al*. 2015). Furthermore, ELF4, a homologue of *At1g72630*:* ELF4‐L2* in QTL 4, has also been implicated in flowering time, where the *elf4* single mutant displays a late‐flowering phenotype (Doyle *et al*. 2002). Another interesting candidate is *At1g30060*:*COP1‐interacting protein* in QTL 1 that has not been directly implicated in flowering time; however, *cop1* displays a late‐flowering phenotype (McNellis *et al*. 1994). Even though their direct role in plant resistance remains to be investigated, these flowering‐related candidate genes suggest that life‐history strategy may affect plant responses to stress.

## Discussion

Plants are exposed to a wide range of biotic and abiotic stresses and need to deal with this ensemble of stresses in order to survive and reproduce. Yet, responses of plants to stress are usually investigated for individual stresses and for individual plant accessions. Plant accessions may differ in life history, which may decisively influence their exposure to a subset of the total set of environmental stresses and thus the selection pressures expressed. Here, we executed an extensive study to investigate whether life history affects the response of 308 European *A. thaliana* accessions to seven different stresses, including both single stresses and combinations of stresses. The most salient result of our study is that life history of *Arabidopsis* plants has a major effect on resistance to these stresses, rather than latitude, longitude or altitude. This is in line with a recent meta‐analysis that shows that genetic variation in life history characteristics has a strong correlation with resistance to stress (Carmona *et al*. 2011). In particular, life history traits such as flowering time exhibited the strongest genetic correlations with herbivore susceptibility (Carmona *et al*. 2011). Our multitrait GWA analysis suggests the involvement of life history strategy‐related genes in plant responses to different types of stresses. These genes are related to the timing of flowering and thus further indicate that genes influencing life history strategy may be involved in plant responses to stress. Because life history strategies such as being summer annual or winter annual have consequences for the plant's exposure to environmental stresses such as drought or herbivory, a link between the two traits may likely have been under positive selection. Which genes underlie the contrasting responses of summer annuals and winter annuals to stresses remains to be experimentally investigated. The fact that there are flowering‐related genes in the 12 QTLs identified should incite the search to their possible involvement. However, it should be realized that a QTL allele effect in an association analysis represents the conditional difference between two groups of genotypes with alternative versions of a SNP and epistatic interactions may play a role (Caicedo *et al*. 2004). Furthermore, our data show that summer annuals of *A. thaliana* are more resistant to stress involving caterpillars, either as single stress or as component of a combination of stresses, whereas winter annuals are more resistant to drought and herbivores such as aphids and thrips. The summer annual is considered the derived state (Michaels *et al*. 2003; Amasino 2004). The present study implies that the evolutionary shift from winter annual to summer annual imposes new selection pressures.

### Geographic patterns in genetic diversity of European accessions

The 308 European *A. thaliana* accessions displayed a strong isolation by distance (IBD) which implies that, relative to distantly located accessions, accessions from the same location are genetically similar. Our findings are in line with several studies that have reported strong similar IBD patterns in *A. thaliana* accessions from Europe (Sharbel *et al*. 2000; Koornneef *et al*. 2004; Ostrowski *et al*. 2006; Schmid *et al*. 2006; Platt *et al*. 2010), although such isolation is weaker among the accessions introduced in North America (Platt *et al*. 2010). Many factors can contribute to isolation‐by‐distance patterns, but *A. thaliana* populations are suggested to become genetically isolated at a fast rate due to the self‐pollinating nature of the species (Alonso‐Blanco & Koornneef 2000). This trait is thought to be also important in explaining why *A. thaliana* populations are highly inbred with large genetic variation within and among populations (Nordborg *et al*. 2005; Horton *et al*. 2012). This kind of population structure has implications for our study, especially when inferring local adaptation. If similar genotypes are clustered in space, correlations between genotype and environment may arise due to spatial clustering and lead to misleading interpretations (Platt *et al*. 2010). For example, Allard *et al*. (1972) provided a classical example of local adaptation in *Avena barbata* in California, but a deeper analysis revealed that these populations were not locally adapted but spatially structured (Latta & Gardner 2009). In our study, we corrected for these potentially misleading associations by implementing a phylogenetic mixed model that accounted for the genetic resemblance among individuals and, therefore, their spatial colocation.

### *Arabidopsis* responses to abiotic and biotic stresses differ between life history strategies

Two life history strategies have been described in *A. thaliana*: winter and summer annuals (Pigliucci 1998; Koornneef *et al*. 2004; Shindo *et al*. 2007). The winter annual is considered the ancestral state because loss and reduced function alleles at two genes (*FRI* and *FLC*) confer the summer annual phenotype (Michaels *et al*. 2003; Amasino 2004). It is generally accepted that summer annuals occur closer to the equator while winter annuals occur in temperate regions (Johanson *et al*. 2000; Michaels *et al*. 2003). Despite this general assumption, no clear geographic patterns were observed for the occurrences of these two life strategies in an earlier study (Shindo *et al*. 2007). Here, we observed a higher proportion of summer annuals at high elevations and southern latitudes. In line with this observation, an increase of summer annuals with increasing altitude in Spain (400–1700 m) and the Swiss alps (600–2700 m) has been reported (Pico 2012; Luo *et al*. 2015). It is interesting that a decrease in grasshopper abundance is correlated with a decrease in resistance of plants to chewing insect herbivores (Descombes *et al*. 2017). This, together with data on the correlation of life history with resistance to herbivory (Carmona *et al*. 2011) further supports the connection of life history strategy and selection pressure by herbivores. The predominance of summer annuals at high elevations has been attributed to the extended and cold winters that *A. thaliana* may be able to withstand as seed but not as rosette. On the other hand, several studies have reported a predominance of summer annuals at low altitudes along elevational gradients in the Iberian peninsula (100–1600 m; Montesinos‐Navarro *et al*. 2011; Mendez‐Vigo *et al*. 2013) that was explained by the extreme drought conditions in summer that may select for early flowering (Luo *et al*. 2015). Furthermore, we also observed genetic clusters consisting mostly of winter annuals at northern latitudes. This is also consistent with literature reports where nonrandom distribution of strong winter annuals has been observed in Scandinavia (Shindo *et al*. 2005).

Independent of the biogeographic distribution of life cycle strategies, our study revealed that life cycle strategy is the most important factor explaining plant responses to most of the stresses studied here, except for resistance to thrips. We found that the degree of damage to thrips (*F. occidentalis*) was strongly influenced by geographic‐genetic structure. This was also reflected by the high heritability values estimated for thrips resistance. Interestingly, this geographic‐genetic structure resembles the geographic‐genetic distribution of glucosinolates, a well‐established defence mechanism against generalist herbivores, including thrips (Zust *et al*. 2012; Brachi *et al*. 2015).

Several authors have suggested that at higher latitudes, herbivore pressure is lower and hence plants are less defended (Pennings & Silliman 2005; Salazar & Marquis 2012; Halbritter *et al*. 2013). Assuming that winter annuals are more common in these habitats, we expected that relative to summer annuals, winter annuals would be less defended against herbivory (Pennings & Silliman 2005; Salazar & Marquis 2012; Halbritter *et al*. 2013). Indeed, we found that winter annuals were more susceptible to damage by the two chewing specialist caterpillars *P. rapae* and *P. xylostella* than summer annuals. However, the opposite was observed for the two piercing‐sucking generalist herbivores: the phloem‐feeding aphid *M. persicae* and the cell‐content‐feeding thrips *F. occidentalis*. Whether this difference between the effects of life history on chewing and phloem‐sucking herbivores is correlated with the differences in feeding style or phenology remains to be investigated. Although we only tested four different insect herbivore species, these results add to the growing body of literature that suggests that the degree of specialization (Mathur *et al*. 2011; Ali & Agrawal 2012; Barrett & Heil 2012) and insect feeding guild (De Vos *et al*. 2005; Bodenhausen & Reymond 2007; Bidart‐Bouzat & Kliebenstein 2011) may exert different selective pressures on plants.

*Arabidopsis thaliana* accessions displaying different life cycle strategies cope with drought stress in a different manner. For instance, winter annuals employ drought avoidance (i.e. mechanisms that maintain the internal water status under limited water conditions such as stomatal closure and increased root growth) and summer annuals employ drought escape (i.e. shift in phenology that allows plants to grow and reproduce by avoiding activity during periods of water scarcity; McKay *et al*. 2003; Des Marais *et al*. 2012; Juenger 2013; Easlon *et al*. 2014). Furthermore, a link has been observed between life cycle strategies and heat tolerance, such that late‐flowering plants are more sensitive to heat stress (Bac‐Molenaar *et al*. 2015). We, therefore, predicted that under our controlled experimental conditions, summer annuals would be less adapted to drought than winter annuals. Our hypothesis was confirmed. When exposed to drought stress, summer annuals gained less weight than winter annuals. A strong correlation between flowering life history and drought resistance has already been observed in the laboratory in *A. thaliana* (McKay *et al*. 2003; Juenger 2013; Easlon *et al*. 2014). In fact, winter annuals have higher water‐use efficiency than summer annuals (Juenger 2013; Lovell *et al*. 2013). The association between these two traits has been suggested to be partially caused by alleles of the *FLC* and *FRI* genes (McKay *et al*. 2003; Scarcelli *et al*. 2007). A recent study that exposed a large collection of *A. thaliana* accessions to controlled moderate drought found the opposite, that is that summer annuals were more resistant than winter annuals (Bac‐Molenaar *et al*. 2016). The discrepancy between these studies may arise from different levels or time patterns of drought stress applied. For instance, it has been determined that severe drought and moderate drought elicit different physiological and molecular responses in *A. thaliana* (Skirycz *et al*. 2011). In addition, studies on other taxa also found evidence that flowering‐time genes can have pleiotropic effects on other traits such as water‐use efficiency in *Brassica rapa* (Franks 2011), vegetative biomass in *A. barbata* (Latta & Gardner 2009) and size at reproduction in *B. rapa* (Haselhorst *et al*. 2011).

Here, we tested the hypothesis that *A. thaliana* responses to stress would vary with elevation, latitude and longitude. The only significant correlation found was between longitude and *A. thaliana* responses to drought: drought resistance (i.e. reduced weight loss when experiencing the stress) increased eastwards. Interestingly, flowering time in European *A. thaliana* accessions has also been found to correlate with longitude, where the proportion of early‐flowering accessions increases eastwards (Samis *et al*. 2008). In the present study, we also observed a longitudinal gradient for flowering time decreasing eastwards. Interestingly, experimental evolution research on several plant species demonstrated that wet soil and late‐season drought conditions selected for early‐flowering accessions which displayed low water‐use efficiency. On the other hand, early‐season drought selected for higher water‐use efficiency (Heschel & Riginos 2005; Sherrard & Maherali 2006). Large differences in physiological and transcriptional changes upon drought stress have been reported between winter and summer annuals in *A. thaliana* (Des Marais *et al*. 2012), further underlining the importance of life history in responses to stress. Future efforts should be devoted to understanding at the mechanistic level how accessions displaying distinct life cycle strategies cope with abiotic and biotic stresses.

### Plant responses against different stresses exhibit trade‐offs

Upon stress, plants are able to elicit defence or resistance mechanisms that are specific for the attacker or adverse abiotic condition (Reymond *et al*. 2000; De Vos *et al*. 2005; Verhage *et al*. 2011; Appel *et al*. 2014; Stam *et al*. 2014). However, plant responses to a specific stress are usually modulated at the individual or population level by co‐occurrence with other stresses, because plants have limited resources and are locally exposed to different selection forces. Thus, the differential responses of summer and winter annuals to different stress types are likely to reflect differential selection pressures experienced by plants exhibiting these two life history strategies. In addition, life history theory predicts that the defensive repertoire is also genetically constrained so that adaptation to different stresses can trade‐off. For example, abiotic stresses tend to have a negative impact on how plants deal with pathogens or herbivores (Suzuki *et al*. 2014; Ramegowda & Senthil‐Kumar 2015). We hypothesized that the different *A. thaliana* accessions would be limited in their responses to stresses so that responses would be negatively correlated. A negative correlation was observed between responses to *P. rapae* herbivory and drought stress or aphid reproduction on the plants. Trade‐offs are expected when the defences are specifically tailored and are costly to the plant (Erb *et al*. 2011). This suggests that responses to drought and *P. rapae* are specific and costly. An example of specific defences that constrain each other is the interaction between jasmonic acid (JA) and salicylic acid (SA) signalling pathways (Sendon *et al*. 2011; Van der Does *et al*. 2013; Caarls *et al*. 2015). Caterpillar feeding and drought induce an overlapping response at the transcriptional level (Reymond *et al*. 2000; Bodenhausen & Reymond 2007; Verhage *et al*. 2011; Vos *et al*. 2013a), yet with clear specific aspects as well (Coolen *et al*. 2016). At the metabolic level, clear differences can be recorded (Weldegergis *et al*. 2015). Recently, it has been shown that JA signalling is required to increase ABA levels under water stress conditions (De Ollas *et al*. 2015) and that ABA is involved in plant defence against caterpillar feeding (Vos *et al*. 2013b). A plausible explanation for the observed trade‐off between resistance to drought and feeding by the specialist herbivore *P. rapae* may be resource allocation. Furthermore, extensive downregulation under drought stress has been observed for genes that are upregulated by *P. rapae* herbivory, suggesting a mechanistic explanation for this trade‐off (Coolen *et al*. 2016).

## Conclusion

We exposed 308 well‐genotyped European *A. thaliana* accessions to diverse biotic and abiotic and combined stresses. We found that plant life cycle strategy is a major determinant of responses to the different stresses that we applied in this study. Moreover, an extensive trade‐off between *A. thaliana’*s response to *P. rapae* herbivory and drought and between the responses to *P. rapae* and aphids was observed. Several putative genes, including several life history strategy‐related candidates, resulted from an MTMM GWA analysis. Future studies should aim to understand the mechanisms of how accessions with different life history strategies deal with different stresses.

N.H.D.O., M.P.M.T., K.J.K. performed experiments. N.H.D.O., E.F., K.J.K., G.G., J.V.H. performed data analysis. N.H.D.O., J.J.A.V.L, M.D., G.G., K.J.K., M.P.M.T conceived the experimental design. F.F.M.B. and J.J.B.K. performed experiments related to flowering time. N.H.D.O., E.F., M.D., J.J.A.V.L., K.J.K., J.J.B.K., J.V.H. contributed to the interpretation of the analysis. All authors contributed to writing the manuscript.

## Data accessibility

All data are available on the Molecular Ecology website.

## Supporting information


**Table S1** Summary of the climate variables mined for this study that vary along geographical gradients.
**Table S2** Bayesian phylogenetic mixed model analysis to assess differences in flowering time without and after vernalization and geographical gradients.
**Fig. S1** Geographic distribution of *Arabidopsis thaliana* accessions from the Hapmap population.
**Fig. S2** Subset of accessions for Europe.
**Fig. S3** Correlation matrix between geography and climate variables for the 308 *A. thaliana* accessions investigated.
**Fig. S4** Experimental Design and Treatments Scheme.
**Fig. S5** Spatial and genetic distribution of summer annual (219 accessions) and winter annual (89 accessions) life histories in *A. thaliana*.
**Fig. S6** Feeding damage (mm^2^) by thrips per genetic clusters.
**Fig. S7** Variables that display a geographical gradient.

**Data S1** Data used for downstream analysis. Columns A&B present the unique identifiers for each accession. Column C represents the genetic groups (See M&M, section: Genetic diversity in European accessions from the Hapmap population). Columns D‐J present the variables that capture the response of each accession to the corresponding stress (See M&M, section: Statistical analysis).

**Data S2** Results from the MTMM GWAs analysis. MAF (Minor allele frequency). PCp1 =  First principal component for traits that were resistant in summer annuals. PCp2 =  First principal component for traits that were resistant in winter annuals. MTMM *P*‐values for a full model, trait specific and common are presented. Details of the different models are explained in the M&M section. Symbols and descriptions were obtained from TAIR 10. Minimum (Min) and Maximum (Max) relative expression values (log2) for plants exposed to jasmonic acid (JA) or salicylic acid (SA) were obtained from http://bar.utoronto.ca/. In red are indicated the associations identified through MTMM while in black are SNPs in LD (r2 ≥ 0.5) in a half window of 20 Kb.
