# Supplementary material for: Natural variation in life history strategy of Arabidopsis thaliana determines stress responses to drought and insects of different feeding guilds
Source: Mol Ecol. 2017 Apr 6;26(11):2959–77. doi: 10.1111/mec.14100 (PMC5485070; doi:10.1111/mec.14100)
Supplement: Supplementary file 1 — Table S1 Summary of the climate variables mined for this study that vary along geographical gradients. Table S2 Bayesian phylogenetic mixed model analysis to assess differences in flowering time without and after vernalization and geographical gradients. Fig. S1 Geographic distribution of Arabidopsis thaliana accessions from the Hapmap population. Fig. S2 Subset of accessions for Europe. Fig. S3 Correlation matrix between geography and climate variables for the 308 A. thaliana accessions investigated. Fig. S4 Experimental Design and Treatments Scheme. Fig. S5 Spatial and genetic distribution of summer annual (219 accessions) and winter annual (89 accessions) life histories in A. thaliana. Fig. S6 Feeding damage (mm2) by thrips per genetic clusters. Fig. S7 Variables that display a geographical gradient. [file MEC-26-2959-s001.docx]

**Natural variation in life-history strategy of *Arabidopsis thaliana* determines stress responses to drought and insects of different feeding guilds**

Nelson H. Davila Olivas^a^, Enric Frago^a^, Manus P.M. Thoen^acd^, Karen J. Kloth^acd^, Frank F.M. Becker^f^, Joop J.A. van Loon^a^, Gerrit Gort^b^, Joost J.B. Keurentjes^f^, J. van Heerwaarden^e^, Marcel Dicke^*a^

^a^Laboratory of Entomology, Wageningen University, The Netherlands

^b^Biometris, Wageningen University, The Netherlands

^c^Laboratory of Plant Physiology, The Netherlands

^d^Plant Research International, Wageningen University and Research Center, The Netherlands

^e^Plant Production Systems, Wageningen University, The Netherlands

^f^Laboratory of Genetics, Wageningen University, The Netherlands

*Corresponding author: [marcel.dicke@wur.nl](mailto:marcel.dicke@wur.nl)

**Key-words:** natural variation, Arabidopsis, life history, biotic stress, drought, herbivory, specialist, generalist, summer annual, winter annual.

**Table S1.** Summary of the climate variables mined for this study that vary along geographical gradients.

| Variable | Resolution | URL | Reference |
| --- | --- | --- | --- |
| Annual aridity | 1km | http://www.cgiar-csi.org | a |
| PET month 3-6 | 1km | http://www.cgiar-csi.org | a |
| Annual relative humidity | 50km | http://www.sage.wisc.edu/atlas/maps.php | b |
| Elevation | 10km | http://www.sage.wisc.edu/atlas/maps.php | c |
| Annual mean temperature | 1km | http://www.worldclim.org | d |
| Max temperature of warmest month | 1km | http://www.worldclim.org | d |
| Min temperature of coldest month | 1km | http://www.worldclim.org | d |
| Annual precipitation | 1km | http://www.worldclim.org | d |

PET = Potential evapotranspiration, Min= Minimum, Max= Maximum.

a Trabucco, A., and Zomer, R.J. 2009. Global Aridity Index (Global-Aridity) and Global Potential Evapo-Transpiration (Global-PET) Geospatial Database. CGIAR Consortium for Spatial Information. Published online, available from the CGIAR-CSI GeoPortal at: <http://www.csi.cgiar.org>.

b New, M.G., M. Hulme and P.D. Jones, 1999: Representing 20th century space-time climate variability. I: Development of a 1961-1990 mean monthly terrestrial climatology. J. Climate. 12, 829-856.

c National Oceanic and Atmospheric Administration (NOAA) and U.S. National Geophysical Data Center, TerrainBase, release 1.0 (CD-ROM), Boulder, Colo.

d Hijmans, R.J., S.E. Cameron, J.L. Parra, P.G. Jones and A. Jarvis, 2005. Very high resolution interpolated climate surfaces for global land areas. International Journal of Climatology 25: 1965-1978.

**Table S2.** Bayesian phylogenetic mixed model analysis to assess differences in flowering time without and after vernalization and geographical gradients. For each variable the posterior mean and 95% credible intervals (in parentheses) are presented. For the fixed effects the Bayesian P-value is also presented, and significance indicated in bold text. Because flowering type was estimated based on flowering time, this variable was excluded from the models of flowering time.

|  | Elevation | | Latitude | | Longitude | | Plant genealogy |
| --- | --- | --- | --- | --- | --- | --- | --- |
|  | PM | *P* | PM | *P* | PM | *P* | PM |
| Flowering time | -0.004 (-0.014 - 0.006) | 0.444 | 0.28 (-0.37 - 1.03) | 0.477 | **0.47 (0.01 - 0.84)** | **0.019** | 8020 (5879 - 10084) |
| Flowering time vern. | -0.001 (-0.002 - 0.002) | 0.888 | **0.20 (0.01 - 0.37)** | **0.024** | 0.02 (-0.10 - 0.13) | 0.777 | 143.82 (41.29 - 268.11) |

Flowering time vern. = Flowering time after vernalization, PM = Posterior mean, *P* = Bayesian P-value. Plant genealogy was included as random effect in the models, thus P-values are not reported.


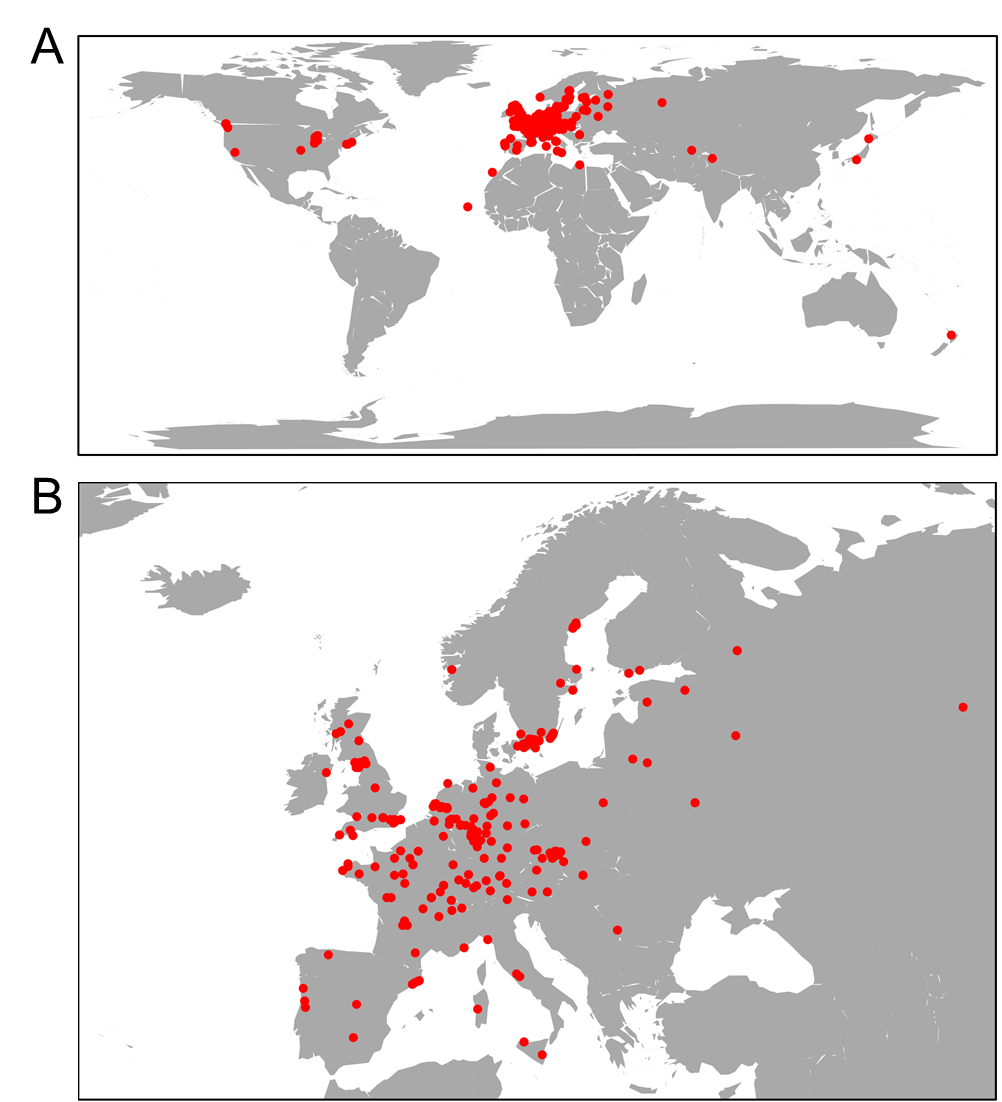


**Figure S1. Geographic distribution of *Arabidopsis thaliana* accessions from the Hapmap population.** (A) Worldwide distribution; (B) European distribution. Locations are shown from which the accessions of the Hapmap population have been collected. The Hapmap population was chosen to represent the geographic range of *A. thaliana*. The plant species is native to Europe and Asia. This native range is represented in the Hapmap population by 320 accessions. Furthermore, *A. thaliana* has been naturalized at many places around the world and this is represented in the Hapmap population by 33 accessions from North America, 2 accessions from Japan, 1 from Libya, 1 from India, 1 from New Zealand, 1 from Cape Verde Island and 1 from the Canary Islands.


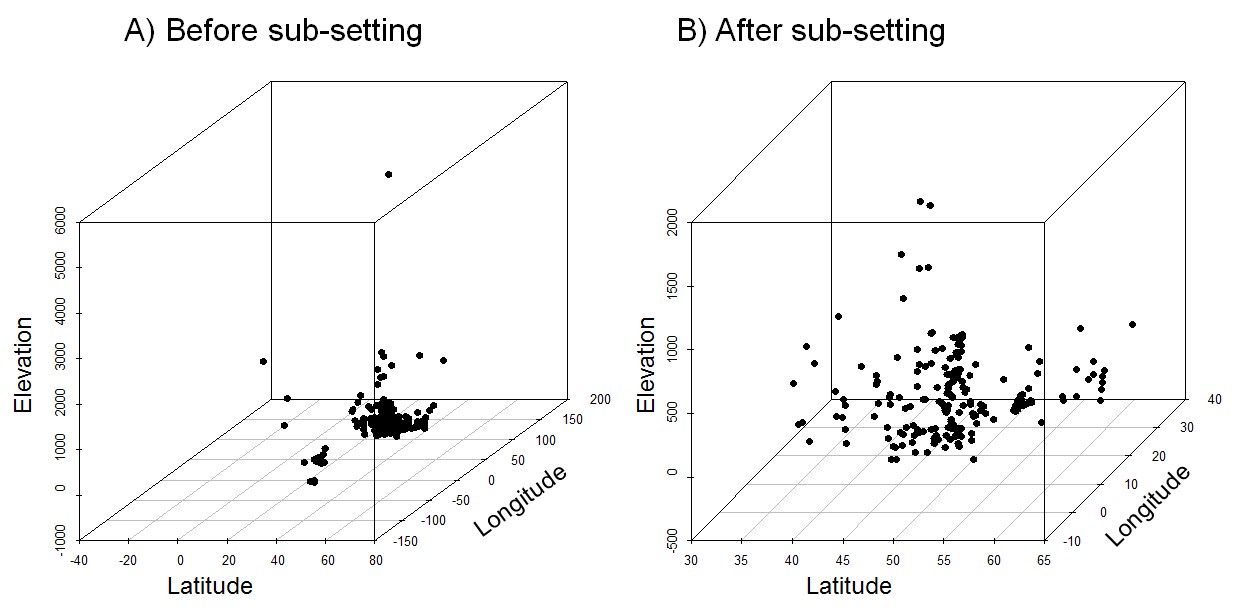


**Figure S2. Subset of accessions for Europe**. We limited the analysis to accessions from Europe (1) To avoid associations to be affected by geographical outliers and (2) Europe represents the centre of genetic diversity in *A. thaliana*, while North American accessions most likely were introduced by humans and are not genetically diverse from Euro-Asia. A) All accessions. B) Accessions limited to a Latitude ≥ 30, Longitude between -50 and 50 and elevation ≤ 2000 m.


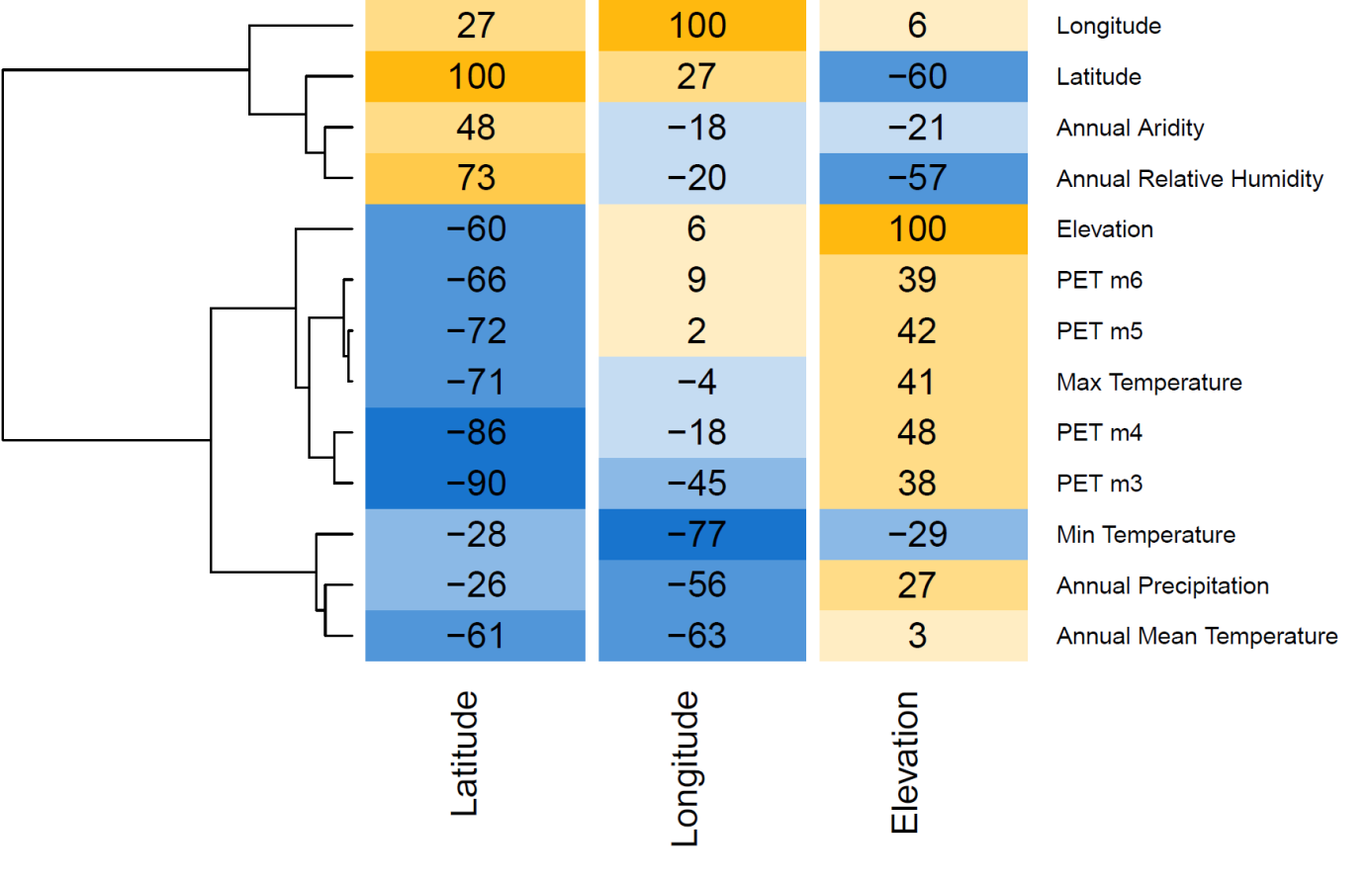


**Figure S3. Correlation matrix between geography and climate variables for the 308 *A. thaliana* accessions investigated.** Heatmap displays Spearman correlation coefficients multiplied by 100. Negative (blue) and positive (gold) correlations are indicated. Climate variables are clustered according to Ward’s minimum variance method. Max temperature = maximum temperature during the warmest month. Min temperature = minimum temperature during the coldest month, PET = Potential evapotranspiration; m 3-6 correspond to the month of the year.


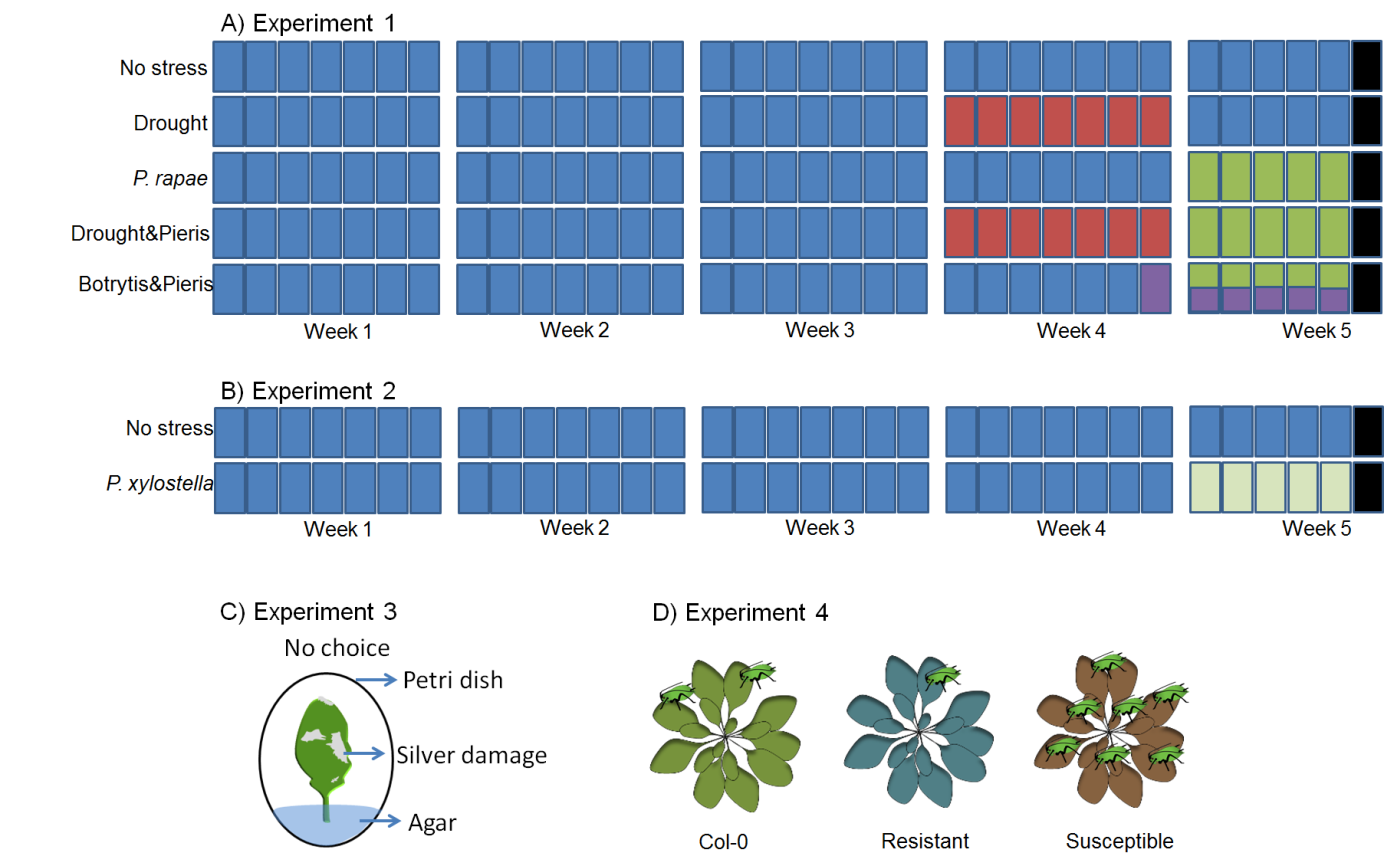


**Figure S4. Experimental Design and Treatments Scheme.** A) In experiment (1) the effects of drought and herbivory by *P. rapae* caterpillars either as single treatment or preceded by drought stress or pathogen infestation were evaluated. Blue indicates that the plants were growing in no-stress conditions. Drought stress period is indicated in red. *Botrytis cinerea* time of inoculation is indicated in purple. *Pieris rapae* inoculation is indicated in dark green. The moment of plant response assessment is indicated in black. B) In experiment (2) the effects of herbivory by *P. xylostella* caterpillars was evaluated. Blue indicates that the plants were growing under no-stress conditions. *Plutella xylostella* time of inoculation is indicated in light green. The moment of plant response assessment is indicated in black. C) In experiment (3) the effects of thrips infestation were evaluated by measuring the amount of feeding damage. In experiment (4) aphid reproduction on the accessions was evaluated.

**
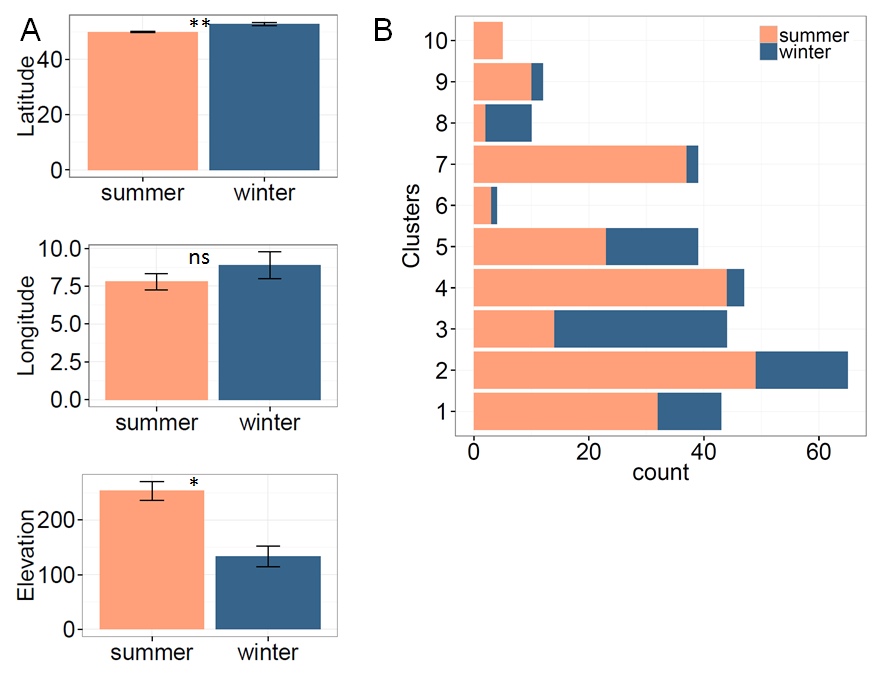
**

**Figure S5. Spatial and genetic distribution of summer annual (219 accessions) and winter annual (89 accessions) life histories in *A. thaliana***. A) Geographical distribution. B) Proportion of summer and winter annuals per genetic group. Bayesian p-values are indicated as ns = *P* > 0.05, * = *P* ≤ 0.05, ** = *P* < 0.01.


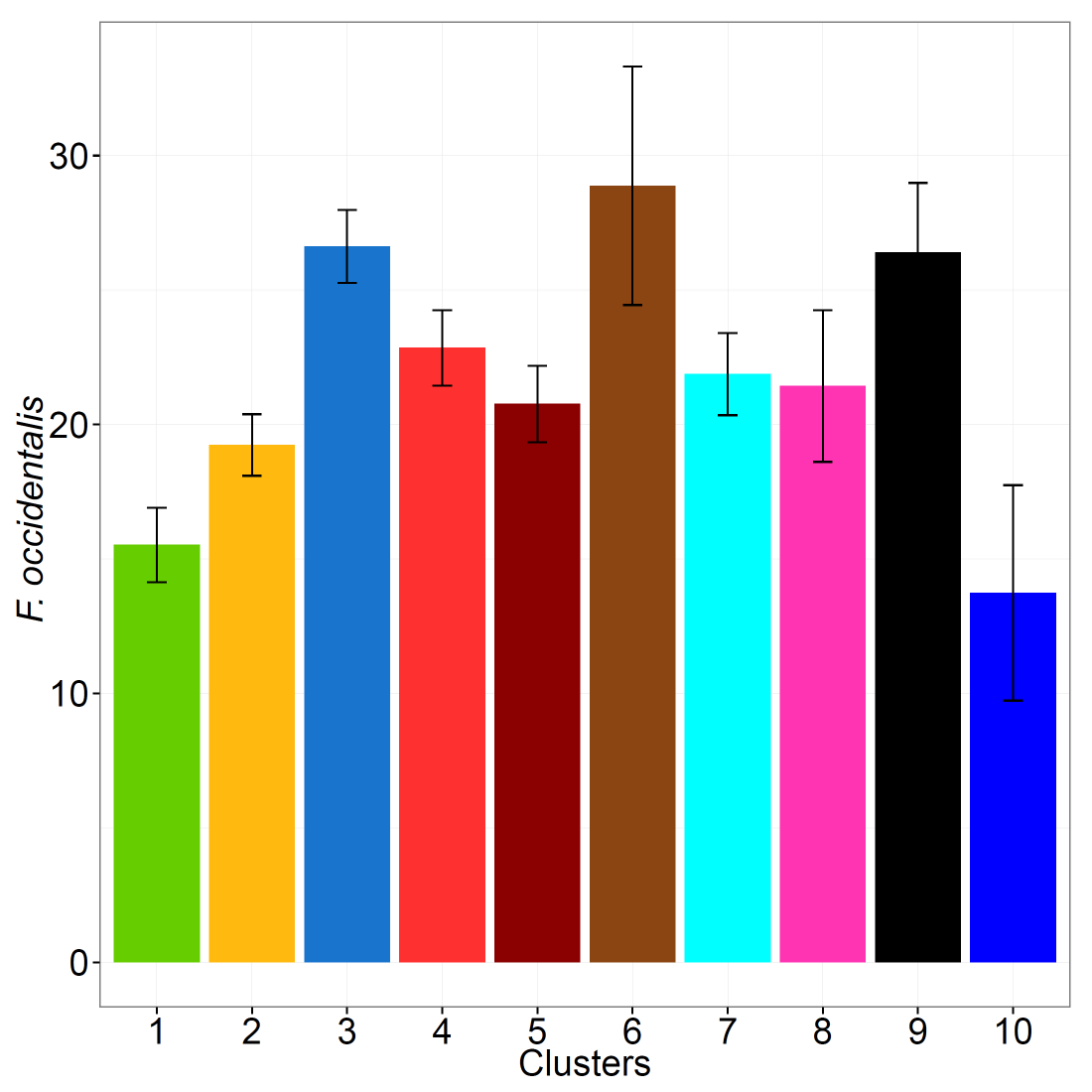


**Figure S6. Feeding damage (mm^2^) by thrips per genetic clusters.** Colours in clusters represent the same colours as in Figure 1 in the main text. Bars show mean value ± SE.

**
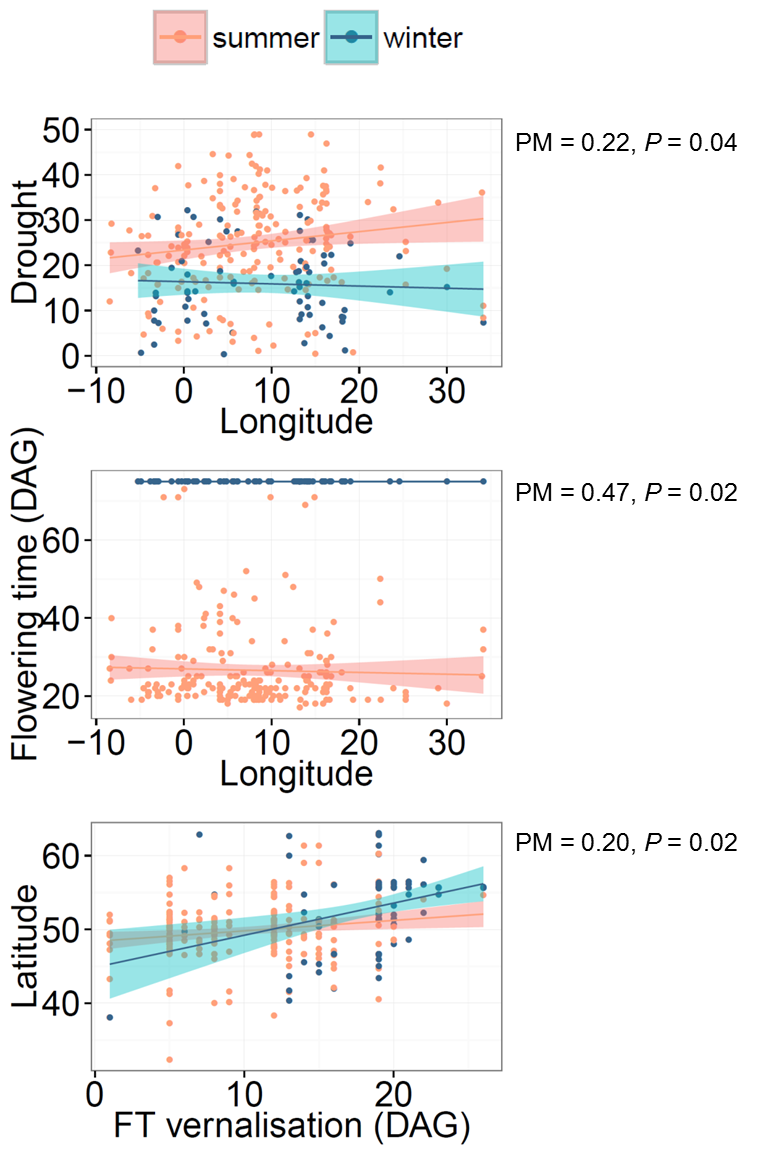
**

**Figure S7. Variables that display a geographical gradient.** The percentage of biomass reduction in *A. thaliana* in response to drought is indicated by Drought. Days to bolting after germination (DAG ) and Days to bolting after a vernalisation period are indicated as Flowering time and FT vernalisation respectively. Posterior mean (PM) and (*P*) Bayesian P-values are indicated. The data on the relationship between latitude and flowering time agree with those by Stinchcomb et al. (2004) for another population.

**Reference**

**Stinchcombe JR, Weinig C, Ungerer M, Olsen KM, Mays C, Halldorsdottir SS, Purugganan MD, Schmitt J. 2004.** A latitudinal cline in flowering time in *Arabidopsis thaliana* modulated by the flowering time gene *FRIGIDA*. *Proceedings of the National Academy of Sciences of the United States of America* **101**(13): 4712-4717.
